# Optimizing Resources for Endovascular Clot Retrieval for Acute Ischemic Stroke, a Discrete Event Simulation

**DOI:** 10.3389/fneur.2019.00653

**Published:** 2019-06-27

**Authors:** Shiwei Huang, Julian Maingard, Hong Kuan Kok, Christen D. Barras, Vincent Thijs, Ronil V. Chandra, Duncan Mark Brooks, Hamed Asadi

**Affiliations:** ^1^The Canberra Hospital, Canberra, ACT, Australia; ^2^Interventional Neuroradiology Service, Department of Radiology, Austin Health, Heidelberg, VIC, Australia; ^3^Faculty of Health, School of Medicine, Deakin University, Waurn Ponds, VIC, Australia; ^4^Interventional Radiology Service, Department of Radiology, Northern Health, Epping, VIC, Australia; ^5^South Australian Health and Medical Research Institute, The University of Adelaide, Adelaide, SA, Australia; ^6^Department of Radiology, Royal Adelaide Hospital, Adelaide, SA, Australia; ^7^Stroke Division, Department of Neurology, Austin Health, Melbourne, VIC, Australia; ^8^Stroke Division, The Florey Institute of Neuroscience and Mental Health, Parkville, VIC, Australia; ^9^Interventional Neuroradiology, Monash Imaging, Monash Medical Centre, Clayton, VIC, Australia; ^10^Department of Surgery and Department of Medicine, Monash University, Clayton, VIC, Australia

**Keywords:** discrete event simulation (DES), endovascular clot retrieval, resource optimization, mechanical thrombectomy, resource allocation, workflow simulation, ECR

## Abstract

**Objective:** Endovascular clot retrieval (ECR) is the standard of care for acute ischemic stroke due to large vessel occlusion. Performing ECR is a time critical and complex process involving many specialized care providers and resources. Maximizing patient benefit while minimizing service cost requires optimization of human and physical assets. The aim of this study is to develop a general computational model of an ECR service, which can be used to optimize resource allocation.

**Methods:** Using a discrete event simulation approach, we examined ECR performance under a range of possible scenarios and resource use configurations.

**Results:** The model demonstrated the impact of competing emergency interventional cases upon ECR treatment times and time impact of allocating more physical (more angiographic suites) or staff resources (extending work hours).

**Conclusion:** Our DES model can be used to optimize resources for interventional treatment of acute ischemic stroke and large vessel occlusion. This proof-of-concept study of computational simulation of resource allocation for ECR can be easily extended. For example, center-specific cost data may be incorporated to optimize resource allocation and overall health care value.

## Introduction

Endovascular clot retrieval (ECR) is the first-line treatment for acute ischemic stroke (AIS) due to arterial large vessel occlusion (LVO) with several trials demonstrating its efficacy in reducing mortality and morbidity (1–3). However, ECR is considerably more costly than traditional care (4), with estimated procedure costs ranging between 9,000 and 14,000 US dollars per patient (4, 5). Major expenditure is required for capital equipment such as angiography equipment purchase and maintenance. Staffing must be adequate to deliver a 24/7 rapid response service.

Government funding agencies seek to optimize return on investment, such as that on resources allocated to acute stroke services. In contrast to other healthcare fields, a resource-use optimization model has not been implemented for comprehensive stroke services. For example, Alvarado and colleagues have identified the optimal staffing capacity to match a service demand increase for an outpatient oncology clinic (6). Luo et al. showed that a decrease in emergency patient wait time for imaging can be achieved by introducing a reserving capacity to the radiology department (7). As healthcare is experiencing both increasing resource demands and fiscal constraints, there is a need to determine how resources may be optimally allocated to improve overall health care value, i.e., high quality and timely care at low cost.

Optimization of resource allocation in healthcare generally involves mathematical or computational modeling of the service pipeline (8). Simulations of outcomes under various competing scenarios identify the most efficient workflow with the least resource usage. Common methods are system dynamics, Markov modeling and discrete event simulation (DES) (9–12). Of these methods, DES is the most widely used approach to optimize healthcare workflow (11, 13, 14). Several features make DES more attractive than the other methods. Firstly, DES allows customizable patient attributes, generating simulations that are patient-focused rather than system or workflow-focused (8, 15–19). Secondly, patient interaction with the environment (i.e., other patients and resources) are tracked in a way that makes it easy to calculate resource usage and patient wait time (8, 15–19). Lastly, empirical comparison between DES and Markov modeling showed that the former approach scales better with model complexity (19). For these reasons, DES is routinely used by industries for workflow optimization and appears to be an appropriate tool for investigating ECR resource utilization.

Here we implemented a model of ECR resource utilization using the open source statistical language R. We developed an interactive web application of a discrete event model of an ECR service, enabling patient wait time and resource utilization to be simulated under various competing scenarios. While the online model was developed based on conditions relevant to our 2017 local health environment, it is widely configurable to model any ECR service.

## Methods

**Figure 1:**
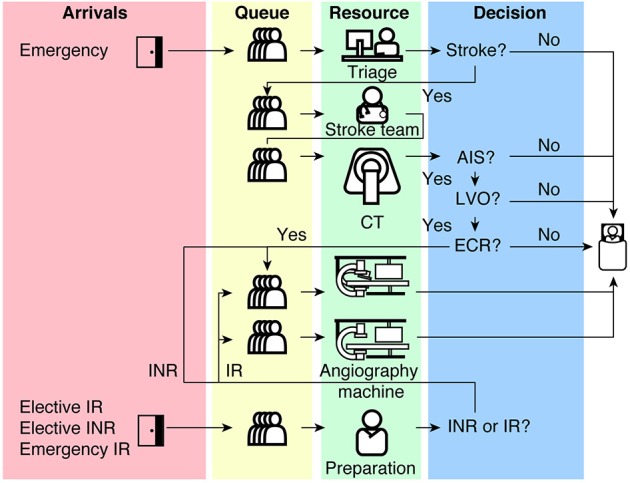
A schematic diagram of our discrete event model of an ECR service from Emergency to angiography suite. CT, Computed Tomography; AIS, Acute Ischemic Stroke; LVO, Large Vessel Occlusion; ECR, Endovascular Clot Retrieval; IR, Interventional Radiology; INR, Interventional Neuroradiology.

A discrete event model describes a continuous workflow process as a sequence or sequences of discrete events (20, 21). Each event is an interaction between a system entity and a workflow resource. In our case, entities represent patients, and resources represent human and physical resources such as interventional radiologist (IR), interventional neuroradiologist (INR), stroke physician, nurse, radiology technologist, CT scanner, single plane (angioIR), and biplane (angioINR) angiography suites. In between events, the model assumes that no changes occur to patient and resource statistics. In this study, the ECR service is described as multiple overlapping sequences of discrete events as illustrated in Figure 1. The source code for the model is available at https://github.com/shiweih/desECR under a GNU General Public License.

### Model Algorithm

Briefly, the model describes four overlapping sequences of discrete events re-presenting (1) a stroke pathway, (2) an elective non-stroke interventional neuroradiology (elective INR) pathway, (3) an emergency interventional radiology (emergency IR) pathway and (4) an elective interventional radiology (elective IR) pathway.

The stroke pathway begins with a new patient in the Emergency Department (ED) and ends with the patient “seizing” an angioINR, an INR and angio staff which represents nurses and technologists. The patient must proceed through a sequence of events chronologically as follows: triage in ED, assessment by the stroke team, CT imaging, assessment for ECR eligibility and lastly, acquiring ECR resources (Figure 1). The decision to proceed to the next event is probabilistic and is acquired from logged data from a Comprehensive Stroke Service in Melbourne, Australia, between 2016 and 17 (Table 1).

**Table 1:** DES model inputs.

**Resource**		***N***	**Capacity schedule**
**(A)**			
Physical resources	Angiography machine for INR and IR	1	Not applicable
	Angiography machine for IR only	1	Not applicable
	CT	2	Not applicable
Human resources	Interventional neuroradiologist	1	24 h
	Interventional radiologist	2 1	0800–1700 1700–0800
	Angiography staff	6 3	0800–1700 1700–0800
	ED team	10	24 h
	Stroke team	1	24 h
**Patients**	***N***	**Average interarrival time (min)**	
**(B)**			
ED	107,700	5	
suspected stroke	750	701	
AIS	450	1,168	
ECR	58	9,062	
elective INR	104	5,054	
emergency IR	468	1,123	
Elective IR	3,805	138	

***(A)** Human and physical resources. **(B)** Patient statistics*.

The elective INR, elective IR and emergency IR pathways are modeled because they utilize resources shared with the stroke pathway. These pathways are each represented by two sequential events: procedure preparation by angiography staff and the procedure itself which is the concurrent use of an angiography suite (angioINR or angioIR), angiography staff and a radiologist (IR or INR).

A distinction is made between elective and emergency patients (including emergency IR and stroke patients). While elective patients specifically compete for resources during work hours, emergency patients require immediate treatment and therefore have priority in resource queues (see next section for details).

### Model Properties

#### Patients

Patients are generated by a Poisson process with an inter-arrival time as specified in Table 1. Inter-arrival times are calculated from patient statistics which were obtained from logged data from a Comprehensive Stroke Service in Melbourne, Australia between 2016 and 17. For example, we estimated that 3,800 elective IR patients were managed by the IR service between 2016 and 17. This translates to an inter-arrival time of 138 min.

#### Events

An event is when a patient interacts with a resource, where an interaction is the patient queueing for a resource, “seizing” or occupying a resource, or releasing a resource. Once a resource is seized, the resource becomes unavailable for other patients to use until its release.

#### Queuing

In the real world, resources are preferentially given to emergency patients over elective or non-emergency patients. In our model, emergency IR and stroke patients have higher priority than elective patients for resources. Specifically, angioINRs are capable of both INR and IR procedures, although all patient types can utilize this resource, stroke patients have priority compared to other patient types. Emergency IR patients are next in line, followed by elective patients. For example, if a stroke patient and an emergency IR patient enter a queue with 10 elective patients for angioINR, the stroke patient will automatically be placed in front of the queue followed by the emergency IR patient. For an angiography machine for IR procedures only (angioIR), emergency IR patients have priority over elective IR patients. When no resources are available, but multiple resource choices are present, a patient automatically enters the resource queue with the least number of entities (i.e., the shortest queue).

#### Capacity Schedule

Capacity schedule describes the maximum number of staff available at a given time of the day. This feature allows us to define staffing numbers during work and after-work hours.

### Outcome Measures

We examined two outcome measures in this model: the patient wait time and resource utilization rate. “Patient wait time” is the time spent queuing for a resource. “Resource utilization rate” represents the median occupancy rate.

### Statistics and Software

The goal of this study was to compare how resource allocation strategies affect patient wait times. Although the DES model provides outputs in terms of numbers of patients waiting at each resource, these numbers can be difficult to compare across simulations and are not inherently meaningful, as they are sensitive to model parameters. To facilitate graphical and descriptive comparison across models, we express waiting times as relative probabilities of waiting a given amount of time, compared to not waiting at all. Since most patients accessed services without waiting, wait time densities could be directly compared across simulations after this normalization. Statistical significance was not computed in this study as all comparisons will be statistically significant given sufficient simulation replicates. Each scenario has a runtime of 365 days and was simulated 30 times. The DES model was built with Simmer (version 4.1.0), a DES package for R (22). The interactive web application was built with R-Shiny.

## Results

**Figure 2:**
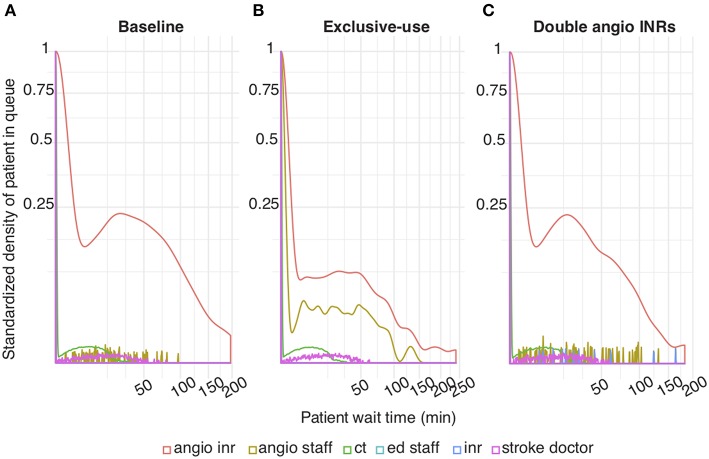
Patient wait time under various simulation scenarios **(A)**. Baseline scenario simulated using inputs from Table 1 **(B)**. Exclusive-use scenario: IR patients can only utilize angioIR **(C)**. Two angioINRs scenario: 2 angioINRs, no angioIRs. Standardized density of patients in queue: the probability density of patients who are waiting standardized to patients who are not waiting.

We simulated a model of an ECR service to identify workflow bottlenecks. Using data from a Comprehensive Stroke Service in Melbourne (Table 1), our simulation showed that the proportion of patients who waited, relative to those who did not, is substantially higher for a biplane angiographic suite (angioINR) compared to other resources (Figure 2A, red compared to other colors).

To investigate why a bottleneck exists at angioINR, we tested three scenarios with varying degrees of patient accessibility to angioINR. First, in the “exclusive-use” scenario, angioINR is not available for elective IR patients. Its use is restricted to stroke, elective INR and emergency IR patients. Second, in the “two angioINRs” scenario, the angioIR is replaced with an angioINR, doubling angiography availability for ECR patients. Lastly, in the “extended schedule” scenario, day time working hours of all human resources are extended by up to 2 h, extending resource access to all patients.

### Exclusive-Use Scenario

In this scenario, the overall wait time probability at angioINR was reduced compared to baseline (red line in Figure 2B compared to Figure 2A). This represents a decrease in ECR patient wait time for angioINR by an average of 6 min. Furthermore, the exclusive-use scenario explains the mechanism underlying the bottleneck at angioINR. While ECR patients have been explicitly programmed to skip the queue for angioINR, they must still wait for this resource to be vacated. However, angioINR is almost never “empty” on patient arrival because it is constantly occupied by other patient types, especially elective IR patients who outnumber ECR patients by a factor of 65 (elective IR: 3805, ECR: 58). Preventing elective IR patients from using angioINR redistributes resources to ECR patients and thereby reduces their wait time.

### Two angioINRs Scenario

This scenario simulates the effect a facility upgrade to two biplane angiographic suites, but without additional staff changes. The wait time probability at angioINR was reduced compared to baseline (Figure 2C). The reduction represents an average of 4 min less in queue for angioINR.

### Extended Schedule Scenario

**Figure 3:**
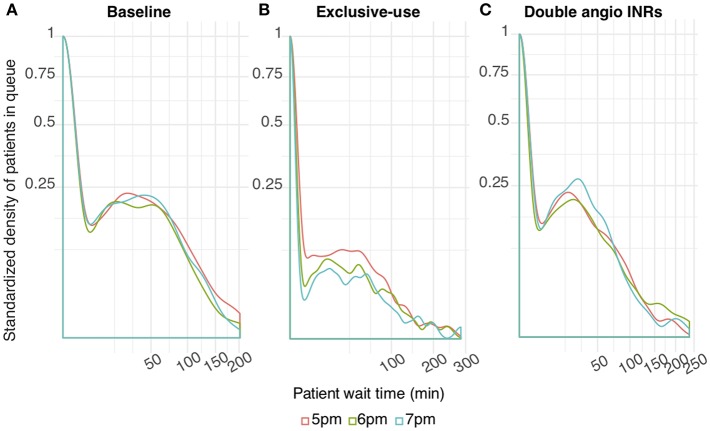
The effect of increasing working hours on ECR patient wait time at angioINR **(A)**. Baseline scenario **(B)**. Exclusive-use scenario **(C)**. Two angioINRs scenario. Standardized density of patients in queue: the probability density of patients who are waiting standardized to patients who are not waiting.

The wait time probability at angioINR in the exclusive-use scenario was further reduced by extended work hours (Figure 3B). In contrast, work extension did not affect baseline or the 2 angioINRs scenario (Figures 3A,C). For the baseline scenario, 1 and 2 h of extra work resulted in an average wait time of 1.7 and 0.9 min reduction, respectively. For the 2 angioINRs scenario, 1 and 2 h of extra work resulted in an average wait time gain of 1 and 0.3 min, respectively.

### Clinical Outcomes of Reducing ECR Patient Wait Times

**Figure 4:**
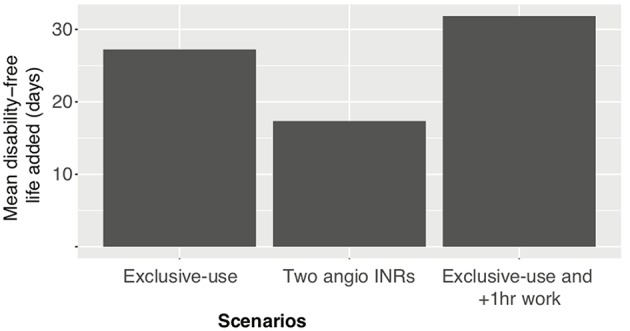
Disability-free life gained under various scenarios.

Reducing patient wait time for ECR has been shown to increase disability-free life at a rate of 4.2 days per minute (23). Based on this rate, the exclusive-use, exclusive-use with 1 h of extra work and the two angioINRs scenario saves on average 4, 4.5, and 2.5 weeks of disability-free life (Figure 4).

### Resource Utilization

**Figure 5:**
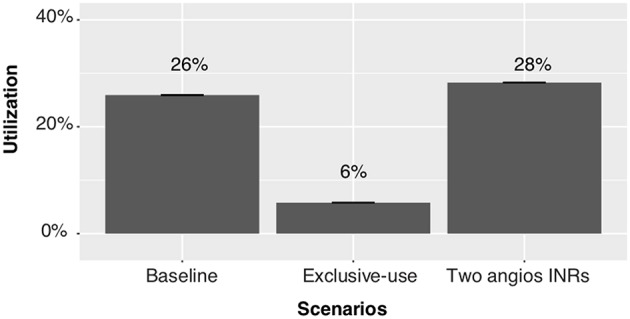
A comparison of the utilization of angioINR by ECR patients under various scenarios.

Whilst the exclusive-use scenario is the most effective at reducing ECR patient wait time for angioINR, the utilization rate of angioINR is the lowest (6% relative to 26% for the baseline scenario, Figure 5). In contrast, no relevant change to the utilization rate of angioINR was observed in the two angioINRs scenario.

### Using DES to Predict Future Resource Usage

Since acquiring data for this study, the demands for ECR at our Comprehensive Stroke Service has doubled between 2018 and 19 and is predicted to triple by the end of 2019. We simulated these increased demands on the resource. As expected, the patient wait times do become longer, but the patterns of resource utilization remained unchanged, suggesting that the same bottlenecks affect throughput (Supplementary Figure).

## Discussion

Our study illustrates the potential use of DES to optimize resource allocation for ECR. Specifically, the ability of DES to model patient-resource interaction allows immediate identification of service bottlenecks and resource underutilization. In brief, the bottleneck at angioINR in the baseline scenario is attributed to resource competition from elective IR patients. Limiting access of elective IR patients to angioINR is an approach to reduce ECR patient wait time, but that is at the cost of straining the elective IR service and underutilizing angioINR.

It is important to note that the absolute values of the model output should not be taken literally as the models are simplifications of a complex multi-disciplinary service. What should be taken into consideration is the dynamic change of the output in response to parameter variation, which is why we represented the outcomes as densities of wait times scaled to the probability of not waiting for a resource.

While increasing the number of resources is often the default approach to an over-subscribed demand, we did not simulate this scenario because it is trivially true. For example, an additional angiography suite coupled with an additional radiologist and appropriate levels of angiography staff will necessarily reduce patient wait times, though at a cost. However, DES modeling may be used for making decisions on whether capacity expansion is cost-effective vs. varying capacity schedules and resource access. Ideally, modeling should reduce unnecessary infrastructure and staffing investments and allow more rational utilization of existing resources.

The quality of the ECR service appears to be robust to important parameters, such as the number of radiologists. The simulation findings apply to ECR services that can be represented by the model in this study. As such, utilization of this model to its maximum capacity requires tailoring the model to local needs, as institutional bottlenecks differ between providers (24). We specifically developed this model using an open source programming language so that the source code can serve as a basis for future model refinement and modification.

Another common solution to increased service demand is increasing staff working hours. Indeed, this approach does decrease stroke patient wait time, particularly by reducing elective patient wait time (data not shown). However, this solution does increase the calculable cost of overtime pay. This strategy may also have less tangible effects, such as increasing staff overwork and decreasing morale, and these factors need to be considered in conjunction with mathematical modeling for policy development.

Patient arrival pattern determines how congested a service workflow will be, and this pattern needs to be better modeled to produce more realistic patient wait time prediction. Therefore, future model refinement should consider the effects of season, public holidays, time of day, and time of week on patient arrival pattern. Ideally, these parameter specifications should be based on empirical data.

In general, a limitation of the current implementation is that few measurements exist to parameterize or validate many aspects of the simulation, because such records are not routinely kept. However, explicitly modeling the workflow can allow administrators to keep track of key parameters and performance, improving the model over time. This data-driven approach combined with adjustment of work schedules may improve the performance of individual centers.

In conclusion, we built a computational model to mimic the workflow of an ECR environment. Potential future developments of the model include automatic cost calculation, modeling the entire stroke service, from transportation to severity triage through to ECR, and adaptation to other time-critical emergency treatment services. The model is currently available online at https://rebrand.ly/desECR11.

## Data Availability

The datasets generated for this study are available on request to the corresponding author.

## Author Contributions

SH and HA designed the study. SH developed the DES model, website and drafted the manuscript. All authors contributed to manuscript revision.

### Conflict of Interest Statement

The authors declare that the research was conducted in the absence of any commercial or financial relationships that could be construed as a potential conflict of interest.

## Supplementary Material

Supplementary Figure Increasing ECR patient volume on service bottleneck. Standardized density of patients in queue: the probability density of patients who are waiting standardized to patients who are not waiting. **(A)** Baseline scenario. **(B)** Doubling ECR patients in baseline scenario. **(C)** Tripping ECR patients in baseline scenario.
