# Supplementary figures and images for: Optimizing Resources for Endovascular Clot Retrieval for Acute Ischemic Stroke, a Discrete Event Simulation
Source: Front Neurol. 2019 Jun 27;10:653. doi: 10.3389/fneur.2019.00653 (PMC6610480; doi:10.3389/fneur.2019.00653)

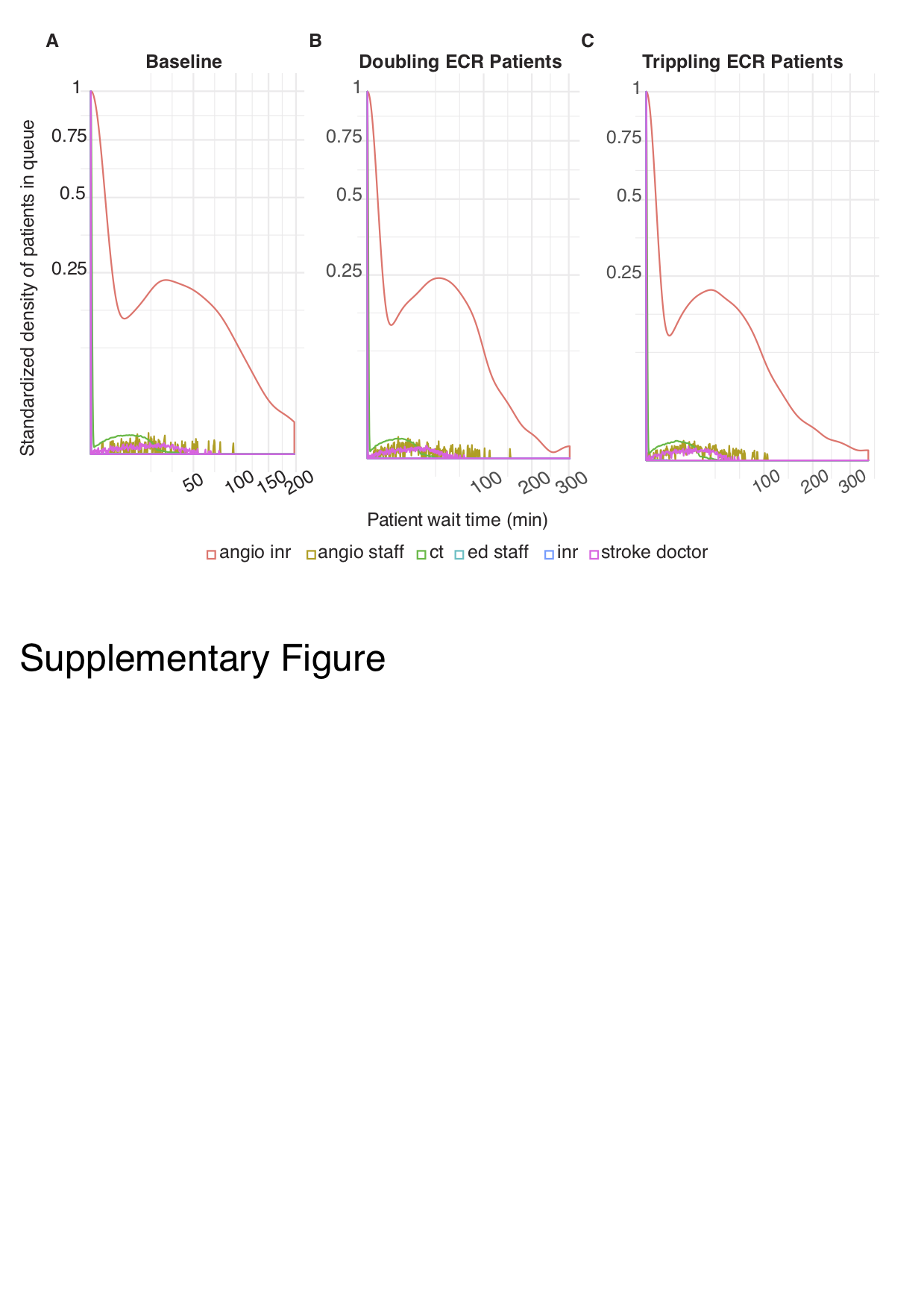

Supplement: Supplementary Figure — Increasing ECR patient volume on service bottleneck. Standardized density of patients in queue: the probability density of patients who are waiting standardized to patients who are not waiting. (A) Baseline scenario. (B) Doubling ECR patients in baseline scenario. (C) Tripping ECR patients in baseline scenario. [file Image_1.TIFF]
